# Comparative Evaluation of Luting Efficacy Between a Dual-Cure and Light-Cure Resin Cement on Its Application on Root Surface Indirect Restorations: An In Vitro Study

**DOI:** 10.7759/cureus.54657

**Published:** 2024-02-21

**Authors:** Twinkle Francis, Karthickraj S M

**Affiliations:** 1 Department of Periodontics, Saveetha Dental College and Hospitals, Saveetha Institute of Medical and Technical Sciences, Saveetha University, Chennai, IND

**Keywords:** health, dental, stereomicroscope, root surface, microleakage, veneers, resin cement

## Abstract

Aim

The aim is to evaluate the degree of dye penetration between a dual-cure and conventional resin cement on its application on the root surface indirect restorations to provide a reference for clinical choice.

Materials and methods

Ten freshly extracted human maxillary central anteriors were selected and randomly divided into two groups of five each. Teeth were prepared for veneer restoration, and veneers were luted using two groups of cements Calibra veneer cement and Fusion Ultra D/C cement. After they were immersed in methylene blue dye solution for 24 h, the specimens were then sectioned buccolingual into three halves in a parallel vertical plane and measured dye penetration using a stereomicroscope (Zeiss). The data collected was recorded by the dye penetration index (0-4) and statistically analyzed using the IBM SPSS Statistics for Windows, Version 24 (Released 2016; IBM Corp., Armonk, New York, United States).

Results

It is evident that group 1 (Calibra veneer cement) showed the lowest mean score of 0.43 mm and group 2 (Fusion Ultra D/C) showed a highest mean score of 0.72 mm. Overall, when the two groups were compared for microleakage using SPSS, there was a significant difference among the groups.

Conclusion

It was determined that both the resin cements evaluated in this study showed microleakage to some level given the limits of the investigation and the findings. To evaluate the dye penetration of microleakage, the Calibra veneer resin cement showed a better marginal adaptability for veneer restoration. Further investigations with broader methodology and more clinical simulation are needed to evaluate other resin cements available for root surface indirect restorations to be analyzed for prospective clinical outcomes.

## Introduction

Due to the growing importance of esthetic appearance, research in restorative dentistry focuses on achieving the expectations of both patients and practitioners. The use of bonded ceramic restorations in dentistry has significantly risen due to the development of adhesive materials that allow for more conservative restorative treatments, as well as the capacity to provide outstanding esthetic appearance and acceptable strength [1]. 

The result of ceramic veneers is largely dependent on the strength and endurance of the bond between the porcelain, luting cement, and enamel/dentin contact, especially when dentin is involved [2]. Because of the thin covering of enamel at this point, it is fairly common for dentin to be exposed, especially in the gingival third of a veneer preparation. Since high failure rates in veneers have been linked to wide exposed dentin surfaces and the cervical margin has been identified as a challenging place to accomplish complete marginal adaptation, the cementation process becomes even more crucial in this instance [2,3]. 

Despite the fact that no restoration or luting material can achieve a full marginal seal, the clinical success of cemented restorations has long been assessed by assessing marginal fit and microleakage. Due to the clinically undetectable passage of bacteria, fluids, molecules, or ions between tooth structure and the cemented restoration, microleakage in the case of all-ceramic restorations has been correlated with the loss of the integrity of the bond to tooth structure and has been linked to other issues like secondary caries, postoperative sensitivity, pulpal inflammation, staining, and plaque accumulation [4-6]. According to reports, the composite resin luting cements experience polymerization shrinkage between 2.6% and 5.7% [7]. It is anticipated that the stress caused by polymerization shrinkage will result in a small space opening between the tooth structure and the ceramic veneer. Two bonded interfaces are created when the ceramic veneer is cemented to the tooth using composite resin cements. One is between the composite resin and ceramic cement, while the other is between the composite resin and the tooth interface. Stress generated by the polymerization and thermal expansion processes causes two bonded interfaces to compete with one another, which causes debonding at the interface where the adhesive strength is lowest. The chemically undetectable movement of bacteria, fluids, chemicals, or ions between the cavity walls and the restorative materials is known as microleakage, and it is one of the key criteria used to evaluate the long-term efficacy of restorative materials [8,9]. An additional proposition is that the variations in the coefficient of thermal expansion (CTE) across bonded surfaces, such as enamel, resin cement, ceramic, and composite, could result in a marginal opening and eventual microleakage due to their dissimilar behaviors under oral heat cycles [10].

Adhesive resin cement systems are a better choice since the advent of modern dual-cure cements, although forming a continuous, durable, and long-lasting interface with the surface of the root is still difficult [11]. The luting cement is intended to solidify into a gap-free mass that perfectly seals the root surface and is stable in the oral environment [12]. Although this is theoretically understood, it is currently unknown whether ready-to-use adhesives offer a hermetic, leak-proof seal. Collagen hydrolysis is a part of the biodegradation of resin-dentin bonds in etch-wash systems, whereas self-etching adhesives are primarily responsible for the hydrolytic degradation at the composite/adhesive interface [13].

The traditional recommendation for luting treatments is the dual-cure resin cements because of their low solubility rate, high mechanical capabilities, and good adhesive qualities [14,15]. Due to their unique synergistic properties with UV-activated (light) chemical cements, dual-cure cements are perfect for treating deep cavities like those seen in root canals. The goal of dual-cure cements can only be satisfactorily achieved if the crucial preparation of the tooth root employing an adhesive method [16] is carried out. Initially applied in three processes, several adhesive solutions were eventually applied in two steps, and most recently, one self-etch step [16,17]. However, research has shown that this property of the acid resinous monomers found in the surface layer of the two-step and self-etching systems can weaken the adhesive binding to root dentin, causing marginal leakage in the future that ultimately results in a poor prognosis for the restoration [18,19]. Recently, companies such as 3M and GC have created self-adhesive resin cements that don't need the dentin to be pretreated. As a result of these cements' lack of an adhesive system, they significantly reduce the number of application stages and cut the length of clinical therapy, which results in a reduction in technique sensitivity by lowering multiple procedural error phases [20,21]. Self-adhesive cements have been shown to react with the hydroxyapatite crystals of the hard tissue in the oral cavity because they include multifunctional phosphoric acid methacrylates [21,22]. Self-adhesive cements, however, have a lesser capacity to adequately diffuse with the underlying dentin, which results in a slight leakage and a poorer adherence between the substance and the surface, claims some research. Since these materials are still quite new, there isn't much knowledge about their adhesive properties. There is lacunae for adhesive properties of these materials with respect to the bonding of the root interface. This study aims to compare the luting efficacy using in vitro analysis of marginal microleakage between the dual-cure and light-cure resin cements on its application on root surface indirect restorations.

## Materials and methods

Tooth preparation

An experimental in vitro study was created to determine the marginal microleakage of two different dental materials used in veneer restoration. This study was done at Saveetha Dental College, Chennai. The Scientific Review Board (SRB) of Saveetha Dental College (SDC) gave its approval SRB/SDC/PERIODONTICS-1821/22/070 to the research protocol.

Sample size was determined by the following formula N= Z^2^ . δ^2^ /(X1-X2)17.16^2^, where Z = 1.96, δ = pooled standard deviation of 0.72, X1 = mean of group 1 (=0.84), and X2 = mean of group 2 (=0.35). Substituting these values in the above formula, a sample size of 9.7 was obtained.

For the investigation, 10 human maxillary anterior teeth that had recently undergone extraction due to periodontal pathology within the previous four months were used. None of the teeth chosen had noncarious cervical lesions, structural abnormalities, prior fillings, caries, or cervical abrasions. Using normal saline, which was known to have no impact on dentinal permeability or the binding strength of cement for this experiment, the calculus and debris were removed by ultrasonic scaling. According to the restorative materials allotted to each group, the teeth were separated into two main groups of five teeth each. Each tooth was prepared for a veneer using the same method. The buccal surface was given a 0.5 mm reduction and the occlusal surface was given a 135° sloping shoulder finish line. The bur was changed after every two tooth preparations.

Veneer preparation

**Figure 1:**
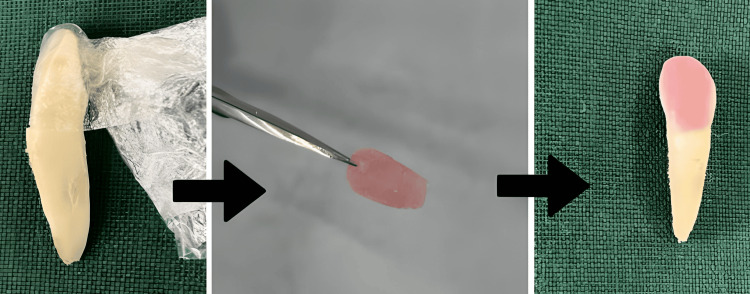
Tooth preparation for veneer placement The prepared tooth surface covered using a cling wrap as a spacer for the luting cement, over which cold cure resin is placed in increments in order to fabricate the veneer. The veneer is then detached and excess was trimmed. Veneer was fixed using the particular luting cement designated to each group of teeth.

**Figure 2:**
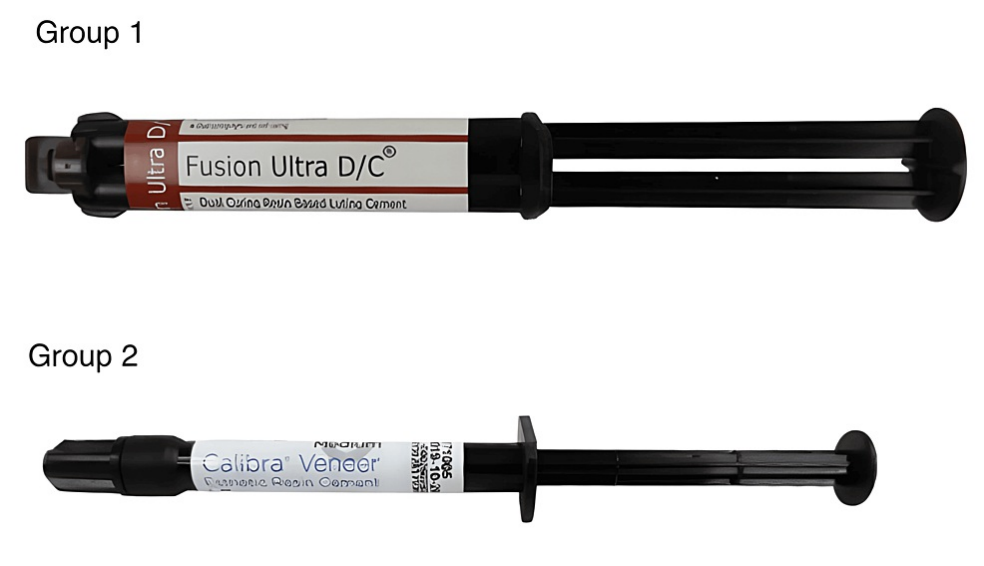
Two groups of resin cements used for veneer luting Teeth belonging to group 1 were designated with Fusion Ultra D/C cement, Prevest DenPro, USA, to be luted and those belonging to group 2 were designated with Calibra veneer cement, Dentsply Sirona, India, to be luted.

Then a cling wrap was used to cover the surface of the preparation to ensure proper equivalent flow of the luting cement below the surface of the veneer to be cemented. The veneer was prepared by using cold cure powder and monomer step by step over the cling-wrapped surface in order to prevent sticking of the cold cure cement onto the natural tooth. After drying, the cold cure veneers were removed from the surface, and the excess was trimmed out (Figure 1). Two resin luting cements were chosen: Fusion Ultra D/C cement, Prevest DenPro, USA; and Calibra veneer cement, Dentsply Sirona, India, based on the curing property (Figure 2). The prepared veneers were then cemented onto all the prepared teeth using the two materials.

Restorative procedure

**Figure 3:**
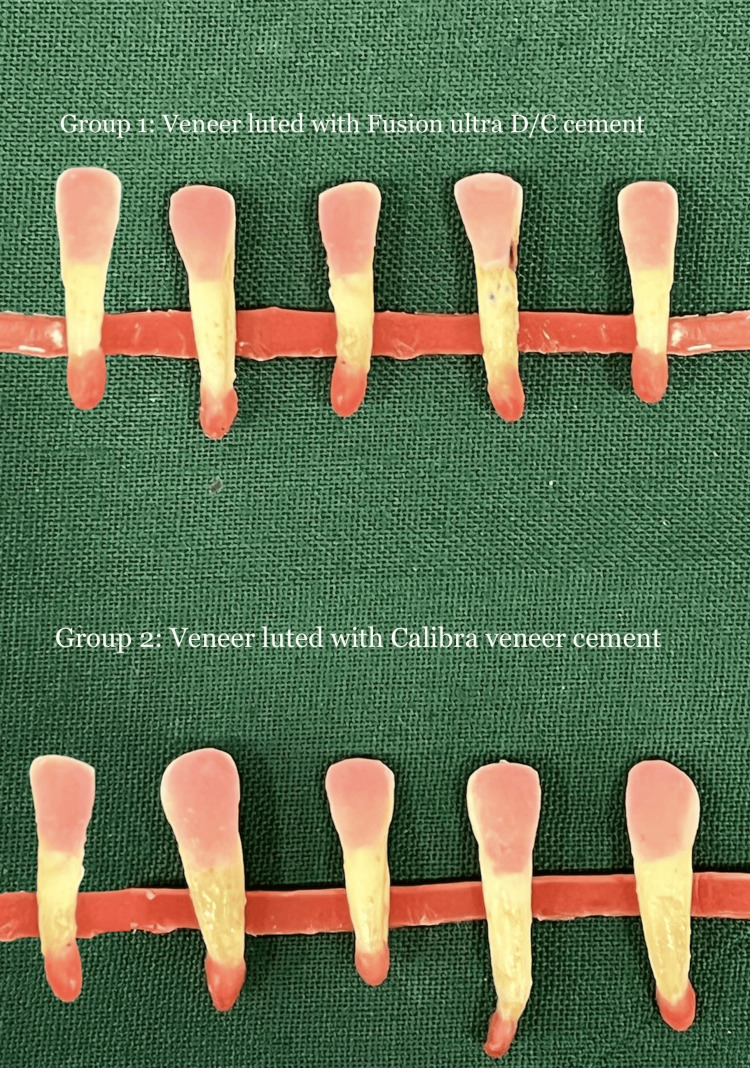
Veneers luted with the resin cement Group 1 veneers were luted with Fusion Ultra D/C cement, Prevest DenPro, USA. Group 2 veneers were luted with Calibra veneer cement, Dentsply Sirona, India.

The intaglio surface of the veneers was coated with resin cement. Each group's teeth were subsequently given veneers, which were firmly secured in place using finger pressure. All of the margins were protected with an isolation gel (Oxyguard, Kuraray) prior to the veneers being exposed to light on all edges for 60 seconds as instructed by the manufacturer (Coltolux 50, Coltene/Whaledent) in order to avoid the formation of an oxygen-inhibited layer (Figure 3). All preparations were performed at room temperature.

Evaluation of microleakage

**Figure 4:**
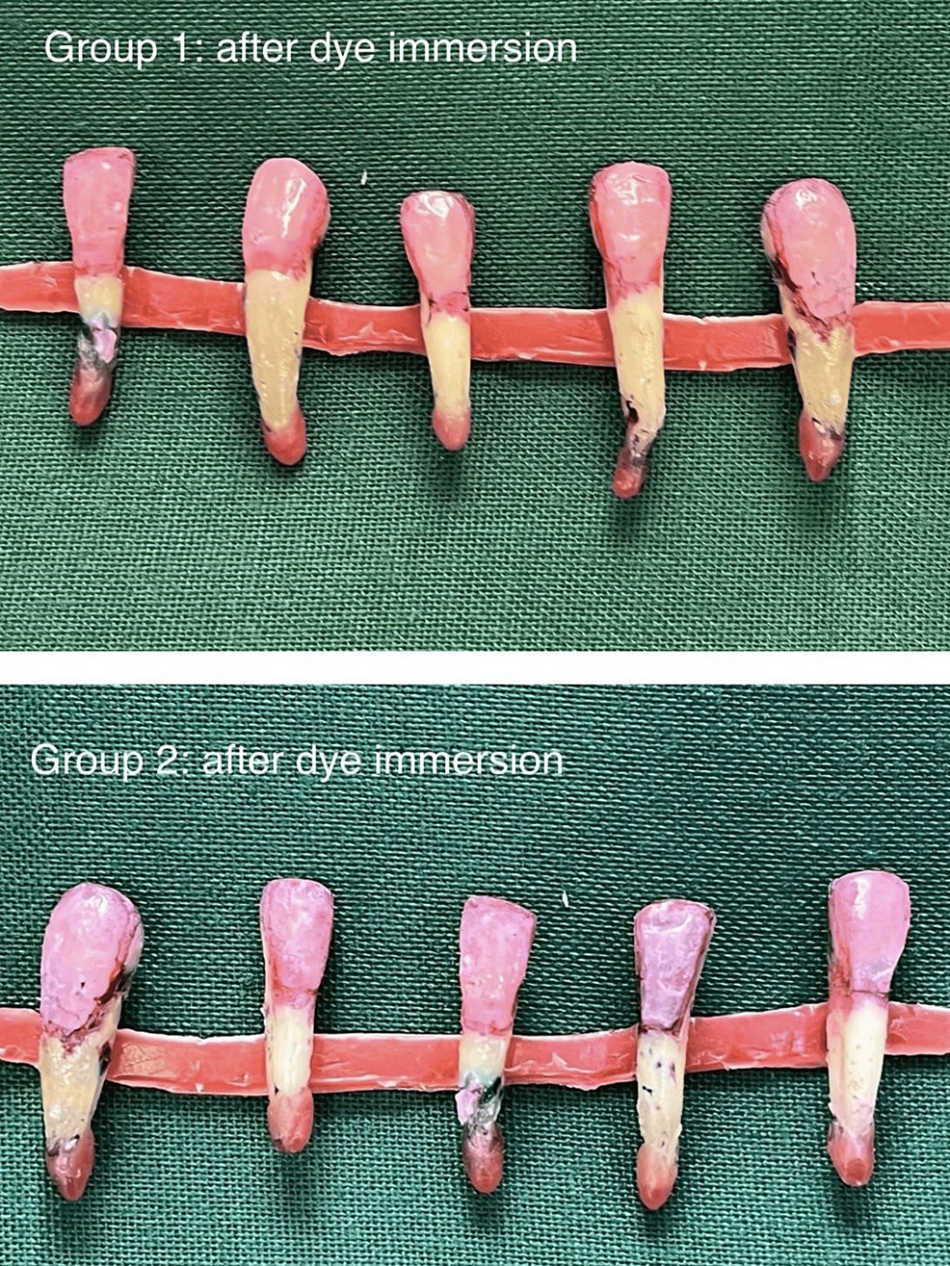
Dye immersion All teeth were washed under tap water after dye penetration and placed in the specific groups.

All teeth had their root apex thoroughly sealed with utility wax prior to the evaluation of the microleakage. Various methods are available for evaluating the microleakage of restorations. Among the two-dimensional evaluation methods, dye penetration was considered to be the most common methodology for assessing microleakage in vitro and provides direct observation of dye penetration. There are several other new newer three-dimensional evaluation techniques such as confocal laser scanning microscopy, microcomputed tomography, and optical coherence tomography [23]. Hence, dye penetration methods were taken for the current in vitro analysis. All teeth, with the exception of the restoration and a 1 mm border of tooth structure surrounding the borders of the restoration, were coated with three coats of clear nail polish to prevent the dye from permeating through the apex or dentinal tubules. The specimens were sealed, fully dried, and then submerged in a methylene blue dye solution for 24 hours. Then, they were taken out of the dye solution, washed under running water, and allowed to dry for an additional 24 hours in accordance with the protocol (Figure 4).

Stereomicroscopy analysis

**Figure 5:**
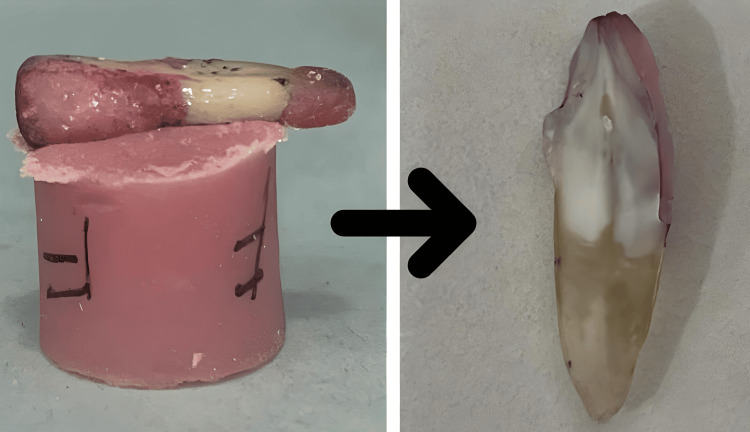
Sectioning done for evaluating the dye penetration Longitudinal section depicts the interface between the tooth and the laminate veneer restoration showing the dye penetration.

The specimens were then set in orthodontic resin blocks before being cut into three equal pieces buccolingually with the use of a diamond disc and a water-cooled diamond saw (Leitz 1600) in a vertical plane parallel to the long axis of the tooth (Figure 5). A single investigator prepared each sample, and two other assessors used a stereomicroscope (Zeiss) at a 40X magnification to objectively evaluate the dye penetration. At a magnification of 40x, a stereo microscope was used to measure the microleakage in each group. The results were recorded using a digital parametric scale in a laboratory. A quantitative number will be provided by the mean leakage of samples from groups 1 and 2.

The Radhika et al. approach was also used to score the microleakage [24]: 0 indicates that no dye has penetrated, 1 that it has only reached the outside half of the axial wall, 2 that it has only reached the inner half of the axial wall, 3 that it has reached the pulpal wall, and 4 that it has penetrated farther than the pulpal wall.

Statistical analysis

The extent of dye penetration data was statistically analyzed using the IBM SPSS Statistics for Windows, Version 24 (Released 2016; IBM Corp., Armonk, New York, United States), and independent sample T test was performed. The p-value was set at 0.05.

## Results

**Figure 6:**
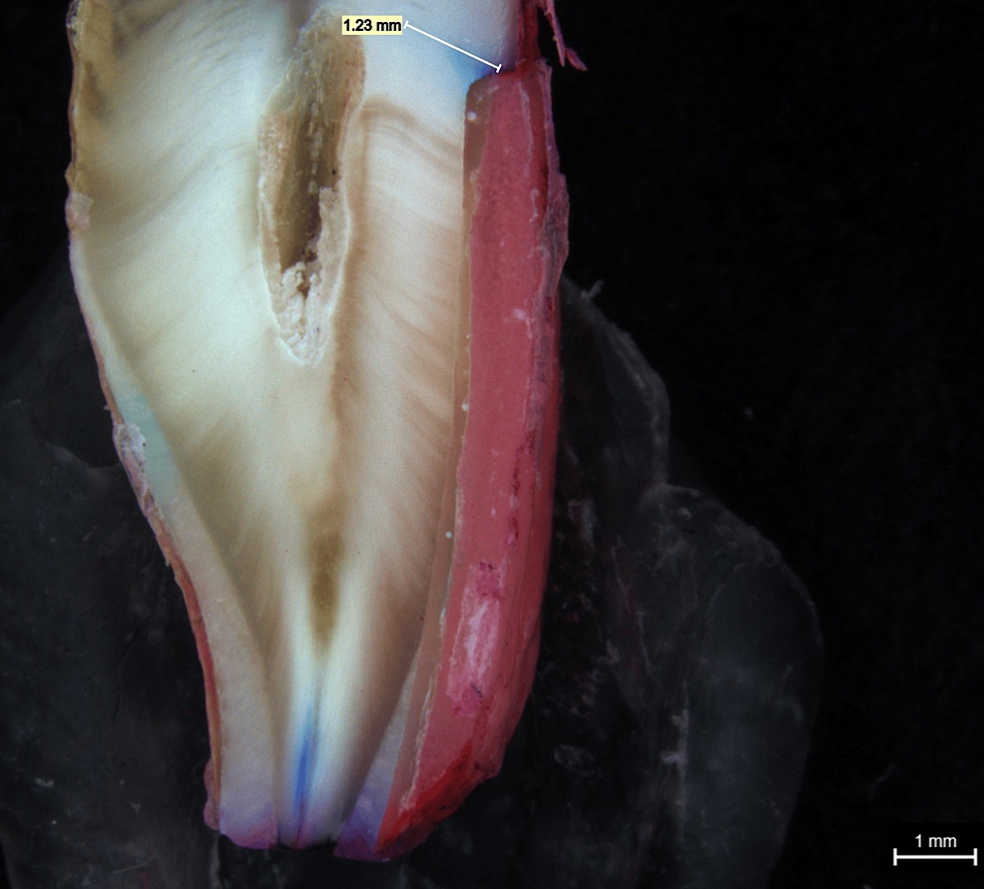
Stereo microscope picture of group 1 dye penetration of a tooth with veneer luted using Fusion Ultra D/C cement

**Figure 7:**
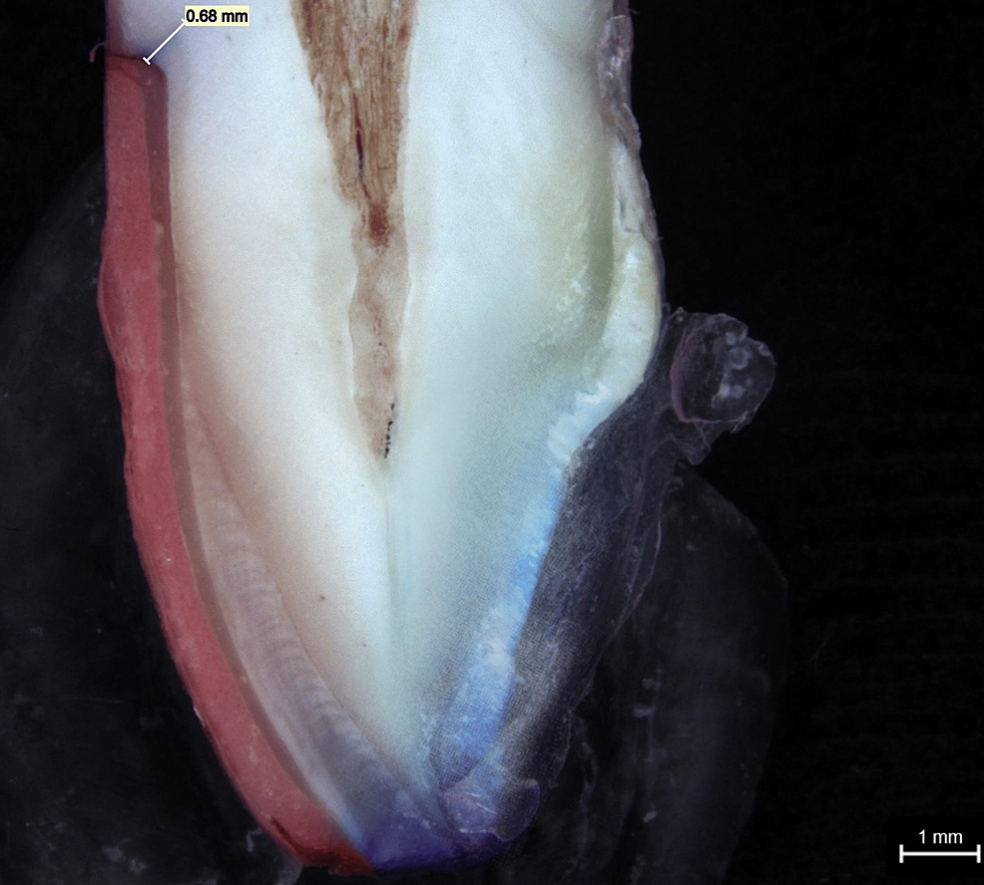
Stereo microscope picture of group 2 dye penetration of a tooth with veneer luted using Calibra veneer cement

Dye penetration measurements observed in a stereo microscope for group 1 and group 2 samples are shown in Figure 6 and Figure 7, respectively. When compared to group 2, which displayed the greatest mean score of 1.63 mm, it can be noted that group 1 displayed the lowest mean score of 0.46 mm. The p-value shows that there was no statistically significant difference between the groups (Table 1).

**Table 1:** The mean extent of dye penetration for group 1 and group 2 resin cements The difference between the groups was statistically insignificant (p = 0.113). N is the number of samples. F is the test statistic of Levene's test, and Sig. is the p-value corresponding to this test statistic.

Groups	N	Mean	F	Sig.
Group 1 (Fusion Ultra D/C)	5	1.63	3.157	0.113
Group 2 (Calibra Veneer)	5	0.46		

**Figure 8:**
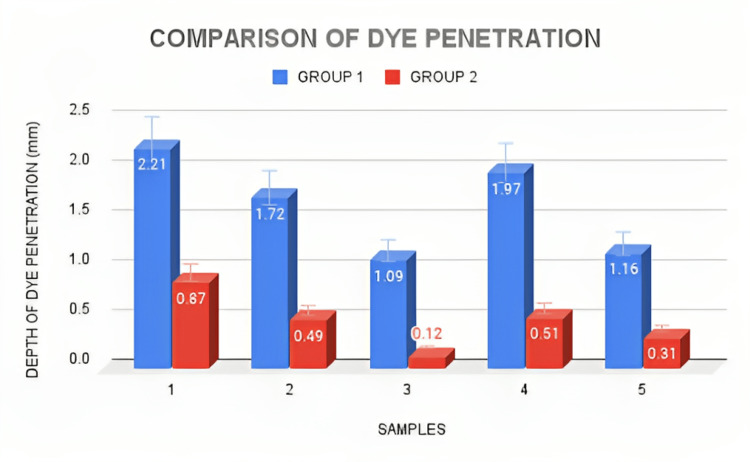
Bar chart depicts the extent of dye penetration seen in both groups of resin cements x-axis represents the samples, and y-axis depicts the extent of dye penetration (mm).

According to the results, the dye penetration extent was higher for group 1 samples than for the group 2 samples (Figure 8). From Table 2 showing the microleakage assessment, three of the samples under group 2 showed a score of 1 and three of the samples in group 1 showed a score of 2 (dye penetration restricted to the inner half of the axial wall) and a score of 3 was observed in group 1 (dye penetration reach the pulpal wall), respectively, while the remaining samples showed a score of 1 (dye penetration limited to the outer half of the axial wall), respectively (Table 2). According to the study results, luting of veneers using group 2 cement showed a mean measurement of 0.46 mm which is lesser than the mean extent of dye penetration seen in luting of veneers using group 1 which had a measurement of 1.63 mm, but it cannot be conclusive as there was no statistically significant difference between the two groups (p > 0.05) (Table 1).

**Table 2:** Scoring of microleakage as per Radhika et al. [23] for the samples used under each group of cement, namely, group 1 (Fusion Ultra D/C) and group 2 (Calibra veneer)

Microleakage scores	0	1	2	3	4
Group 1 (Fusion Ultra D/C)	0	1	3	1	0
Group 2 ( Calibra Veneer)	1	3	1	0	0

## Discussion

In this study, marginal adaptability was also a confounding factor since the conventional veneer materials were not used and cold cure resin materials were used for fabricating the veneers. Resin cements' sealing ability of this veneer material was analyzed with microleakage analysis in the current study. The mean of the dye penetration depth was more with group 1 when compared with group 2 resin cements. The degree of the microleakage observed with scoring also showed that group 1 resin cements had larger scores compared to group 2 resin cements. These results depicted that group 2 resin cements had comparatively better marginal seal without any leakage allowing for good luting efficacy which is not in accordance with another study by Shafiei et al. [25] where the study gave a conclusion that self-adhesive resin cements displayed lesser microleakage and better sealing capability toward dentin.

Schenke et al. conducted a study and compared the mean extent of microleakage in three resin cements and found that the self-adhesive resin cement (RelyX U200) had the best marginal seal with the least microleakage [26]. These results are correlated with our study proving that self-adhesive resin cements were better than both light-cure and dual-cure resin cements. The results of another investigation [27] showed a significant difference between dual-cure resin cements and light-cure and self-adhesive resin cement in microleakage analysis. Evidence was conclusive that dual-cure resin cements had lesser microleakage scores. A study by Pierre et al. [28] assessed the influence of two preparation techniques on microleakage, marginal fit, and cement thickness of lithium disilicate veneers and found that there was a significant difference in microleakage at the cervical area than the proximal area of the veneers but that does have any influence on microleakage or marginal fit.

Several methodologies for detecting microleakage have been discussed in previous studies [23,29], including the dye leakage method, the use of color-producing microorganisms, the air pressure approach, neutron activation analysis, electrochemical research, analysis using scanning electron microscopy, and thermal or mechanical cycling. In this study, the dye leakage method was selected because it provides the direct observation under microscopy, and some degree of microleakage was observed in both cases. The cement from group 2 showed much less microleakage and significantly better results for sealing the margins. Additionally, compared to margins placed on the enamel and dentin surface, the root surface of the current study's restorations was reported to have lesser microleakage when compared to the study by Ibarra et al. which showed that self-adhesive resin cements bond well to the dentin [30]. Thus, problems related to microleakage remain a dilemma to dental practitioners during the choice of luting cement used and may risk the longevity of the restorations.

Even though the study was performed using a limited sample size, in a laboratory setting, the results obtained showed that microleakage can be controlled by the choice of cement in each case. Further tests can be done to evaluate various cements as this was a pilot study. This study design also had limitations with material selection as acrylic veneers and limited resin cements were used for the experimental analysis. 

In future perspective, experimentation should continue exploring for methods to eliminate or, at the very least, limit microleakage due to inefficacy of different resin cements because this may interfere with the proper polymerization of resin composite and mainly poor prognosis in the future causing secondary caries leading to pulpitis manifestation. Three-dimensional invitro analysis and in vivo analysis need to be done in future studies.

## Conclusions

It is determined that both the resin cements evaluated in this study show microleakage to some level given the limits of the investigation and the findings. This is inevitable regardless of the resin cement being used, and in root surface indirect restorations, the microleakage was comparatively lesser in the Calibra veneer resin cement group compared to the Fusion Ultra d/c resin cement group. To evaluate the dye penetration of microleakage, the Calibra veneer resin cement showed better marginal adaptability for veneer restoration. Further investigations with broader methodology and more clinical simulation are needed to evaluate other resin cements available for root surface indirect restorations to be analyzed for prospective clinical outcomes.
